# Literacy and numeracy: Global and comparative perspectives

**DOI:** 10.1007/s11159-020-09854-x

**Published:** 2020-08-12

**Authors:** Anke Grotlüschen, Richard Desjardins, Huacong Liu

**Affiliations:** 1 Universität Hamburg, Hamburg, Germany; 2 Department of Education, University of California Los Angeles (UCLA), Los Angeles, CA USA

The overall aim of this special issue is to contribute to the international discourse around literacy, numeracy, adult education and basic education. It engages with numeracy and mathematical literacy, New Literacy Studies, adult education, and lifelong learning in the context of the United Nations Sustainable Development Goals (SDGs), both from theoretical perspectives and from an empirical viewpoint.

Education affects people’s lives in ways that go far beyond what can be measured by labour market earnings and economic growth. Education contributes to a wide range of social outcomes such as better health, higher levels of civic and social engagement, as well as addressing other socially relevant domains of concern, such as crime, anti-social behaviour and poverty (Schuller and Desjardins 2007).^1^ In the midst of the ongoing global pandemic, individuals who suffer the most, economically, psychologically and socially, are those who are the most disadvantaged in accessing quality education. The COVID-19 pandemic has amplified the need to take a more holistic approach towards education and learning than merely emphasising skills for employability.

The broader approach towards education and learning and the concept of sustainability are already embedded in the history of the United Nations Educational, Scientific and Cultural Organization (UNESCO). They are reflected in the instrumental role UNESCO has played since the end of the Second World War in expanding the right to education to include adults. The call within the fourth Sustainable Development Goal (SDG 4) to “ensure inclusive and equitable quality education and promote lifelong learning opportunities for all” (UN 2015)^2^ has awakened hope among many for a stronger role for adult education in global education agendas and policies (Elfert 2019).^3^ Among the targets within SDG 4,^4^ the one which is of particular relevance to this special issue is SDG target 4.6:

By 2030, ensure that all youth and a substantial proportion of adults, both men and women, achieve literacy and numeracy (UN 2015, SDG target 4.6).

Despite the collective efforts among various stakeholders to achieve progress in working towards the targets of SDG 4, several problematic issues remain, even as we are already five years into implementing the United Nations 2030 Agenda for Sustainable Development. The current pandemic arrived on top of these. The challenges involved in developing indicators to monitor progress were already well-acknowledged at the start of the SDG agenda in 2015. In 2016, led by the UNESCO Institute for Statistics (UIS), the Global Alliance to Monitor Learning (GAML) was established (UIS 2017).^5^ Its mandate is to support national strategies for learning assessments and develop internationally comparable indicators related to SDG 4. GAML set up thematic task forces^6^ which hold expert meetings in order to collect and evaluate existing tests and findings and discuss adequate testing instruments. However, most of the challenges facing GAML and its associated task forces have not yet been resolved and were actively debated at the last GAML meeting in August 2019.^7^

The challenges pertaining to SDG target 4.6 include the complexity of measuring functional literacy and numeracy, the feasibility of reporting adults’ literacy and numeracy against a global common measurement scale, and the difficulty of increasing data coverage among those UNESCO Member States that do not currently have direct assessments in place. GAML’s task force on SDG target 4.6, chaired by the UNESCO Institute for Lifelong Learning (UIL) and the Organisation for Economic Co-operation and Development (OECD), recommended the use of specific skills levels associated with the OECD Programme for the International Assessment of Adult Competencies (PIAAC) as benchmarks.^8^ Specifically, the task force recommended that the minimum proficiency level that should be achieved – and thus count as progress towards SDG indicator 4.6.1 – should be the literacy and numeracy skills level associated with PIAAC Level 1 for high-income countries, and below PIAAC Level 1 (at the sentence-processing level) for middle- and low-income countries (UIS 2018).^9^

These recommendations were based on results of a global consultation as well as analyses of existing population data on skills such as PIAAC and the World Bank’s Skills Towards Employment and Productivity (STEP) study. Empirical data have revealed that approximately 50 per cent of adult populations living in middle-income countries, such as, for example, Turkey and Chile, are at or below PIAAC Level 1 on the literacy scale (OECD 2017).^10^ Therefore, a common benchmark for global reporting purposes could lead to a majority of countries having a large percentage of their adult population classified as being below target levels of literacy proficiency. Arguably, this would make it difficult to assess progress for a large number of countries. However, the Technical Cooperation Group (TCG) on the Indicators for SDG 4^11^ – another committee established in 2016 – recommended using the PIAAC Level 2 descriptor as a reference point for global reporting of SDG indicator 4.6.1 (UIS 2019).^12^

Notwithstanding these challenges, five years into the SDG agenda, the research community has produced methodological advancements that can help measure adult literacy and numeracy at the lower end of the proficiency scale. It has also produced useful knowledge towards understanding literacy and numeracy competences and practices among men and women, albeit mostly in high-income countries. Moreover, scholars have provided important critical perspectives for better understanding the diversity and contextualisation of literacy and numeracy practices. With ten years remaining until the deadline of the United Nations 2030 Agenda for Sustainable Development, it is timely for the *International Review of Education – Journal of Lifelong Learning* (*IRE*) to take stock and gather perspectives from various research fields in order to support a more comprehensive understanding of key issues and challenges surrounding SDG target 4.6 on adult literacy and numeracy.

## Part 1: critiques of the monitoring and measurement of adult literacy and numeracy

The first part of this special issue contains two articles that provide critiques of the monitoring and measurement of adult literacy and numeracy as well as three others that add to the critiques by discussing methodological advancements.

Several scholars have warned that the time pressure and lack of scientific reflection associated with large-scale assessments bear the potential danger of leading to monopolist effects of a handful of education assessment companies or institutions on low- and middle-income countries (Hamilton et al. 2015).^13^ Monitoring the SDGs effectively probably requires a diversity of approaches that exceed what can be achieved by an international and comparative survey like the OECD PIAAC study (Addey 2017).^14^ Unfortunately, other major measurement instruments such as UNESCO’s Literacy Assessment and Monitoring Programme (LAMP) and the World Bank’s STEP study, are closely related to PIAAC and its predecessors, namely the International Assessment of Literacy Skills (IALS) conducted in the 1990s and the Adult Literacy and Lifeskills (ALL) Survey carried out in the early 2000s.

In particular, the test items developed for IALS^15^ and ALL^16^ were designed for contexts derived from high-income OECD countries and were conceptualised exclusively in European languages written in the Roman alphabet. While the LAMP^17^ and the STEP^18^ studies focused on middle- and low-income countries with a wider array of language families and scripts, there is still a lack of sound empirical evidence on how one set of reading components can be used to compare proficiencies across languages and writing systems. Even in cases where such comparability can be arguably established, merely comparing the averages of countries’ literacy and numeracy scores against a common scale or an OECD average risks overlooking the myriad of micro (individual-level), meso (group-level), and macro (government-level) factors contributing to high- versus low- performances (Boeren 2019).^19^ Researchers commented in a recently published article (Grotlüschen and Buddeberg 2020)^20^ that in this kind of global benchmarking, measurements developed in and for countries sharing basic commonalities, such as those belonging to the Western world, are understood and projected as *normality*.

Even though the SDGs explicitly try to overcome the *Brandt Line*^21^ and its North-South division (Singh 2019),^22^ there is a danger that this general application of “normality” to all contexts might become a reality. Therefore, it seems necessary to revisit some underlying theoretical assumptions of measurement and monitoring on a sociological level and from the perspective of educational sciences.

The first article we present in this special issue is entitled “Prophets, saviours and saints: Symbolic governance and the rise of a transnational metrological field”. Arguing from the Bourdieusian perspective of a “sociology of numbers”, *Sotiria Grek* investigates the SDG 4-related monitoring procedures carried out by supranational organisations. Her purpose is to offer insights into the labour and infrastructure involved in the joint production of metrics. Drawing on declarations, agreements and reports as well as empirical findings from a series of interviews she conducted with key actors from major international organisations and civil society, Grek suggests that quantification has facilitated symbolic governance of the education policy field. As a result, the joint effort towards achieving the targets of SDG 4 represents the rise, and to a large degree the dominance, of the influence of the transnational field of measurement in education.

In our second article, entitled “Doing competence: On the performativity of literacy and numeracy from a post-structural viewpoint”, *Lisanne Heilmann* looks at literacy and numeracy from a post-structuralist perspective. This perspective relies on theories of a *relational subject*, as introduced by Judith Butler from a feminist standpoint (Butler 2004).^23^ Heilmann questions the individualised understanding of literacy and numeracy as abstract competences which people simply “have” and explores the possibility of viewing these basic competences as *constructed* through how they are *actively performed* (e.g. when someone engages in reading, writing or calculating for a particular purpose in a particular context) and *referred to* (e.g. when someone is pronounced “literate” or “competent”). She points out that measuring competences implies an individual that “has” competences. What if we are “doing competences”? Emphasising discourse analysis, e.g. by Michel Foucault and Judith Butler, Heilmann elaborates a shift from the New Literacy Studies, which questioned the individualised understanding of literacy as an abstract competence, to a post-structuralist literacy theory.

Challenges from discourse theory and sociology of numbers do not only apply to literacy research, but also to numeracy. Slowly, but steadily, numeracy research is becoming more visible, but adult numeracy can still be seen as a neglected field (Gal et al. 2020).^24^ Being numerate means being critical (Geiger et al. 2015)^25^ – this is especially empowering in the era of “fake news”, when data literacy has become more important than ever. The theoretical concept of *numeracy* shifts towards a more holistic approach and asks for criticality. This seems highly relevant in times of infection statistics and disastrous financial markets, and thus in times where statistics and infection rates inform policymakers and the public on whether to cut back fundamental rights. Therefore, it is most important to discuss how we conceptualise “numeracy” in large scale assessments.

The next article, “Evolution of adult numeracy from quantitative literacy to numeracy: Lessons learned from international assessments” by *Dave Tout,* explains the most recent concept underpinning the ongoing PIAAC cycle, in which relevant changes include the aforementioned critical approach. Data collection is scheduled for 2021–2022 and the results are due to be published in 2023. Tout points out that the development and ongoing refinement of the theoretical frameworks and constructs that shape programmes such as PIAAC and the assessments themselves, alongside the research based on the rich data of empirical and background information emerging from these surveys, have contributed significantly to our knowledge and understanding of numeracy in people’s lives.

However, measurement and monitoring on a global level produce a need for scholarly knowledge on testing especially at the *lower end* of the hierarchically arranged skills levels. Even if many consider a hierarchical model as problematic (Duckworth and Tett 2019; Thériault 2019),^26^ in the absence of any alternatives, these levels will probably still be used for some time around the globe for comparison. However, testing at the lower end of hierarchical scales is problematic due to a lack of testable items and needs competence descriptions. This is addressed in our fourth article, entitled “Proficiency level descriptors for low reading proficiency: An integrative process model”. *Tabea Durda, Cordula Artelt, Clemens M. Lechner, Beatrice Rammstedt* and *Alexandra Wicht* provide a process model based on reader-related, text-related and task-related factors along different stages of the reading process that can cause reading difficulties. Their model enables the identification of *difficulty-generating factors*, in particular task and text characteristics. This model is also suitable for developing proficiency level descriptors by differentiating between a *low* reading proficiency level and a *functional* reading proficiency level among adolescents and adults.

PIAAC has five hierarchically organised proficiency levels for literacy. A sixth category, labelled “below Level 1”, lumps together low proficiencies at the bottom end of the proficiency continuum. While PIAAC Levels are already broadly suitable for international comparison, the in-depth assessment of “Level 1” and “below Level 1” has so far only been focused on by individual countries (e.g. Canada, the United States, the United Kingdom, France and Germany) using instruments developed nationally. Focusing on the reading aspect of literacy, the article which concludes the first part of this special issue investigates how these nationally developed low proficiency assessment instruments might be adjusted to facilitate international comparability.

In “International assessment of low reading proficiency in the adult population: A question of components or lower rungs?”, *Anke Grotlüschen, Barbara Nienkemper* and *Caroline Duncker-Euringer* discuss two competing approaches. One is the *reading components approach,* and the other is the *lower-rungs approach*. Reading components test sets are well-known and widespread. Some of them have been administered in national surveys, some were applied under LAMP in several languages, and many countries opt to run the reading components module of the PIAAC programme. However, starting from IALS in the 1990s, the concept of a hierarchical ladder of proficiency levels has emerged and since been applied with a focus on the lower rungs in the Skills for Life surveys (conducted by the UK Department for Business, Innovation and Skills) and the two German Level-One (LEO) surveys conducted by the University of Hamburg. Based on the German PIAAC Reading Components dataset, Grotlüschen et al. investigate whether the existing component items can be arranged hierarchically (as lower rungs of the ladder) in a similar way as PIAAC and LAMP items to facilitate global reporting and comparison. They conclude that that it is indeed technically possible to integrate the full set of PIAAC reading component test items into hierarchical “rungs”.

## Part 2: findings from qualitative and quantitative literacy and numeracy research

In the second part of this special issue, we present five articles that relate to findings from qualitative and quantitative literacy and numeracy research, and help to reveal several insights and nuances relevant to measurement and monitoring of literacy and numeracy.

Several major theoretical approaches have shown that the discussion of “competences” is shifting towards the notion of “practices”. Social anthropologist Jean Lave, *grande dame* of numeracy research, showed the importance of practices in 1988 with her work on “cognition in practice” (Lave 1988).^27^ Later PIAAC theoretical framework, led by Iddo Gal, built on Lave’s work (Gal et al. 2009).^28^ Most recently, results from longitudinal studies show the impact of practices on competences (Reder 2017).^29^

We begin this second part with an article entitled “Practice makes perfect: Practice engagement theory and the development of adult literacy and numeracy proficiency”. *Stephen Reder, Britta Gauly* and *Clemens Lechner* present more recent findings generated by PIAAC-L, a longitudinal national follow-up survey conducted in Germany, and discuss the influences of practices on competences and vice versa.^30^ Based on *practice engagement theory* (PET) Reder et al. suggest that literacy training which increases engagement in meaningful practices might generate proficiency growth. The authors’ comparisons of how various practice engagement indexes predict growth of literacy and numeracy proficiencies indicate that reading engagement is the strongest predictor of literacy growth, and maths engagement is the strongest predictor of numeracy growth. They conclude their article by considering their findings’ implications for sustainable development, lifelong learning policy and future research into the development of adult literacy and numeracy proficiency.

In our next article, entitled “Micro and macro drivers affecting adult literacy proficiency profiles across countries”, *Richard Desjardins* offers a retrospective of thirty years of assessment with a particular focus on the trend data made available from IALS and PIAAC. The aim of his research is to understand the determinants of literacy proficiency in terms of (1) how they may be affecting the development of literacy from an individual lifecycle (micro-level) perspective, and (2) how they may be affecting the development of national (macro-level) profiles of literacy proficiency as countries’ sociodemographic compositions, sociocultural practices and economies change over time. He discerns an interesting decline of literacy practices in work-related contexts. Possible reasons for this may be that early measurement parameters do not fit any more, or that earlier practices (like looking up something in a printed dictionary) no longer apply in a digital world, but it may also be due to less practical use of skills in everyday contexts, which in the long run can affect skills levels as well. Overall, the decline of literacy practices may be a reason for stagnation in literacy competences (Desjardins 2017).^31^

The eighth article we present in this special issue is entitled “It’s not what you know but where you come from: Cognitive skills, job autonomy and latent discrimination of ethnic minorities in Israel”. Based on PIAAC data, *Sabina Lissitsa* and *Svetlana Chachashvili-Bolotin* investigated the association between cognitive skills and job autonomy among Israeli-born Jews, Arabs and immigrants from the former Soviet Union (FSU) living in Israel. Job autonomy – employees’ freedom to schedule and organise their work independently according to their own experience and preferences – is a major factor in job satisfaction. However, it is not granted to many employees in Israel, and the authors’ close examination of the Israeli labour market reveals that while cognitive skills are positively correlated with job autonomy among Israeli-born Jews and partially among Arabs, these effects are insignificant among FSU immigrants. Lissitsa and Chachasvili-Bolotin analyse their findings through social homophily theory, which explains bonding tendencies among socially and culturally similar people.

Outside the PIAAC measurement industry, questions and findings on literacy and numeracy research extend beyond the question of predictors and outcomes of competences. Based on her narrative interviews with vulnerable adult learners, *Doria Daniels* looks at how adult education and training (AET) learners navigate second-chance education. In her article entitled “Exploring adult education and training as a transformative learning space for alienated out-of-school youth in South Africa”, she investigates what facilitates these learners’ educational success. Education policies in South Africa recently introduced a shift in the function of AET from providing opportunities for the acquisition of literacy to offering a formal qualification. This changed status of AET created a second-chance educational opportunity for youthful, non-traditional “new-generation” adult learners with a troubled history of formal schooling both to complete their general education and/or to further their education and their chances of entering the workforce. Daniels shows how vulnerable second-chance learners slowly, but steadily find ways to advance their quest for an education as energies within AET contexts and their personal worlds come together. Daniels’ narrative interviews also demonstrate how desperate the situation is in South Africa for adults with low literacy skills. Moreover, migration and globalisation call for basic education and pathways to vocational education for immigrants and people with learning disabilities. Adults with learning disabilities – a problematic terminology anyway – are often excluded and overlooked by large-scale assessments and by educational policies.

We conclude this special issue with an article by *Wiebke Curdt* and *Silke Schreiber-Barsch* entitled “Abilities in the blind spot of testing regimes: Eliciting the benefits and the limitations of participatory research approaches for numeracy in adult basic education”. The article argues that adults with *learning difficulties* (also referred to by some as *intellectual disabilities*) and their numeracy-related abilities are still hidden in the blind spot of large-scale testing regimes. Based on rich qualitative data, Curdt and Schreiber-Barsch discuss how participatory research designs can work out in exploring how these learners apply *numeracy practices* in everyday life contexts, and what challenges occur. This allows both a methodological perspective as well as a focus on vulnerable subpopulations (Grotlüschen et al. 2019)^32^ in the field of adult numeracy research. Curdt and Schreiber-Barsch aim to demonstrate benefits and limitations of using participatory research approaches and give evidence why they consider them to be useful for diminishing blind spots of testing regimes.

## Summary

In sum, the authors who have contributed to this special issue reflect on the complexity of literacy and numeracy assessments, focus on the need for a contextualisation of literacy and numeracy practices, and present us with the knowledge that we can gain from analysing existing data generated through large-scale skills assessments over the past 30 years. In particular, they provide critical considerations regarding monitoring and measuring literacy and numeracy, and remind us of those learners who might have been excluded in the policy debates around literacy and numeracy improvement. Between them, these ten articles cover a rich and controversial set of approaches, a set that avoids easy answers or recipes, and instead offers a critical discourse that matches the aims of the United Nations Agenda 2030 SDGs.

Yet, as editors and authors, we are aware of many gaps we could not possibly fill. In particular, it is worth noting that these articles were written before the outbreak of the COVID-19 pandemic. Thus, the timing of this special issue did not allow us to reflect upon the impact of the pandemic on adult learning and education in general, and the monitoring and reporting for SDG target 4.6 in particular. For instance, the short-term impact of school closures when it comes to the ability to read and write, may, in the long run, have a more severe impact on lifelong learning. As commented by Per Magnusson in his blog post published by UIL on 22 May 2020,

Children who do not learn to read early enough often fail later in school or when they enter the labour market: Close a school today and you will end up with an increased share of illiterate adults (Magnusson 2020).^33^

As countries and the international community are joining efforts to fight the pandemic and ensure that learning does not stop for children, young people and adults alike, we are hoping to see sound research on the impacts on adult learning and basic education. We would do well to advance the common goal set by SDG target 4.6.

## Footnotes

1 Schuller, T., & Desjardins, R. (2007). *Understanding the social outcomes of learning*. Paris: OECD.

2 UN (United Nations). (2015). Sustainable Development Goal 4: Ensure inclusive and equitable quality education and promote lifelong learning opportunities for all, targets and indicators [dedicated webpage]. *Sustainable Development Goals knowledge platform* [online resource]. Retrieved 30 June 2020 from https://sustainabledevelopment.un.org/sdg4.

3 Elfert, M. (2019). Lifelong learning in Sustainable Development Goal 4: What does it mean for UNESCO’s rights-based approach to adult learning and education? *International Review of Education*, *65*(4), 537–556. 10.1007/s11159-019-09788-z.

4 For a list of SDG 4 targets and indicators, visit https://sustainabledevelopment.un.org/sdg4#targets [accessed 29 June 2020].

5 UIS (2017). *Global Alliance to Monitor Learning (GAML) Concept paper*. Montreal: UNESCO Institute for Statistics. Retrieved 25 May 2020 from http://gaml.uis.unesco.org/wp-content/uploads/sites/2/2018/10/gaml-concept_paper-2017-en2_0.pdf.

6 For more information about GAML’s task forces, visit http://gaml.uis.unesco.org/task-forces/ [accessed 30 June 2020].

7 Interested readers can get a glimpse of these related debates by reading past GAML meeting documents at http://gaml.uis.unesco.org/sixth-meeting-of-the-global-alliance-to-monitor-learning/ [accessed 30 June 2020].

8 For descriptions of PIAAC literacy and numeracy proficiency, see OECD (2013). OECD (2013). *The Survey of Adult Skills: Reader’s companion,* Paris: OECD. http://dx.doi.org/10.1787/9789264204027-en.

9 UIS (2018). *GAML Taskforce 4.6 Progress Report 2018.* GAML5/REF/4.6.1-12. Montreal: UIS. Retrieved 25 May 2020 from http://gaml.uis.unesco.org/wp-content/uploads/sites/2/2018/12/4.6.1_12_Taskforce-4.6-Progress-Update-Report-UILpdf.

10 OECD (2017). OECD Indicators: Table 2, SDG Targets 4.6, 4.7, 4.a, 4.b, 4.c. In *Education at a Glance 2017* (p. 38). Paris: OECD. https://dx.doi.org/10.1787/eag-2017-table6-en.

11 According to its own dedicated webpage, “The Technical Cooperation Group on the Indicators for SDG 4 – Education 2030 (TCG) serves as a platform to discuss and develop the indicators used for monitoring the Education 2030 targets in an open, inclusive and transparent manner” (http://tcg.uis.unesco.org/ [accessed 30 June 2020]).

12 UIS (2019). *Sixth Meeting of the Technical Cooperation Group on the Indicators for SDG 4 – Education 2030 Summary of decisions and TCG next steps.* Paris: UNESCO. Retrieved 25 May 2020 from http://tcg.uis.unesco.org/wp-content/uploads/sites/4/2019/11/Post-TCG6-Report-Final.pdf.

13 Hamilton, M., Maddox, B., & Addey, C. (Eds) (2015). *Literacy as numbers: Researching the politics and practices of international literacy assessment*. Cambridge: Cambridge University Press.

14 Addey, C. (2017). Golden relics & historical standards: How the OECD is expanding global education governance through PISA for Development. *Critical Studies in Education*, *58*(3), 311–325. 10.1080/17508487.2017.1352006.

15 Countries that participated in the IALS survey were Canada, the Netherlands, Switzerland, Germany, Poland, the United States, Ireland, Sweden, Australia, Great Britain, Northern Ireland, Belgium (Flemish), New Zealand, Chile, Finland, Norway, the Czech Republic, Hungary, Slovenia, Denmark, Italy and Switzerland (Italian-speaking region).

16 Countries which participated in the ALL survey were Bermuda, Canada, Italy, Norway, Nuevo Leon (Northern Mexico), Switzerland, the United States, Australia, Hungary, the Netherlands and New Zealand.

17 The LAMP study was conducted in ten countries including El Salvador, Kenya, Mongolia, Morocco, Niger, Palestinian Autonomous Territories, Vietnam, Jordan, Afghanistan and Lao PDR.

18 Countries that have participated in STEP are Albania, Armenia, Azerbaijan, Bolivia, Bosnia & Herzegovina, Colombia, Georgia, Ghana, Kenya, Kosovo, Lao PDR, Macedonia, Serbia, Sri Lanka, Ukraine, Vietnam, and Yunnan Province in China.

19 Boeren, E. (2019). Understanding Sustainable Development Goal (SDG) 4 on “quality education” from micro, meso and macro perspectives. *International Review of Education*, *65*(2), 277–294. 10.1007/s11159-019-09772-7.

20 Grotlüschen, A., & Buddeberg, K. (2020). PIAAC and the South: Is Southering the new Othering? Global expansion of dominant discourses on adult literacy. *European Journal for Research on the Education and Learning of Adults*, *11*(2), 167–181. 10.3384/rela.2000-7426.rela9214.

21 The “Brandt Line” is named after former German Chancellor Willy Brandt. He chaired an independent commission in 1980 to discuss global development issues and to overcome a view of a First/Second versus Third World. The commission suggested the notion of visual a line depicting the socio-economic and political divide between the Global North from the Global South. based on GDP per capita.

22 Singh, S. (2019). New horizons for the development education in the context of Sustainable Development Goals. *International Journal of Research in Social Sciences*, *9*(3-1), 326–341

23 Butler, J. (2004). *Undoing gender*. New York, NY: Routledge.

24 Gal, I., Grotlüschen, A., Tout, D., & Kaiser, G. (2020). Numeracy, adult education, and vulnerable adults: A critical view of a neglected field. *ZDM Mathematics Education*, *52*(3), 377–394. 10.1007/s11858-020-01155-9.

25 Geiger, V., Goos, M., & Forgasz, H. (2015). A rich interpretation of numeracy for the 21st century: A survey of the state of the field. *ZDM Mathematics Education, 47*(4), 531–548. 10.1007/s11858-015-0708-1.

26 Duckworth, V., & Tett, L. (2019). Transformative and emancipatory literacy to empower. *International Journal of Lifelong Education*, *38*(4), 366–378. 10.1080/02601370.2019.1574923. Thériault, V. (2019). “If you write poems, it’s like a crime there”: An intersectional perspective on migration, literacy practices, and identity curation. *International Journal of Lifelong Education*, *38*(4), 406–419. 10.1080/02601370.2019.1597933.

27 Lave, J. (1988). *Cognition in practice: Mind, mathematics and culture in everyday life.* Cambridge: Cambridge University Press.

28 Gal, I., Alatorre, S., Close, S., Evans, J., Johansen, L., Maguire, T., Manly, M., Tout, D. (Eds) (2009): *PIAAC numeracy. A conceptual framework.* OECD Education Working Paper No. 35. Paris: OECD Publishing.

29 Reder, S. (2017). *Adults’ engagement in reading, writing and numeracy practices.* Applied Linguistics Faculty Publications and Presentations series, vol. 22. Portland, OR: Portland State University. Retrieved 30 June 2020 from https://pdxscholar.library.pdx.edu/ling_fac/22.

30 These data were first presented at the Hamburg Numeracy Project conference held in November 2018, with keynote presentations by Jean Lave, Iddo Gal, Richard Desjardins and Stephen Reder.

31 Desjardins, R. (2017). *Political economy of adult learning systems: Comparative study of strategies, policies and constraints*. London: Bloomsbury Academic,

32 Grotlüschen, A., Buddeberg, K., Redmer, A., Ansen, H., & Dannath, J. (2019). Vulnerable subgroups and numeracy practices. How poverty, debt and unemployment relate to everyday numeracy practices. *Adult Education Quarterly*, *69*(4), 251–270. 10.1177/0741713619841132.

33 Magnusson, P. (2020). COVID-19: Building a sustainable and just future for all. *Only connect, the lifelong learning blog from UIL,* 22 May [blog post]. Retrieved 30 June 2020 from https://thelifelonglearningblog.uil.unesco.org/2020/05/22/covid-19-building-a-sustainable-and-just-future-for-all/.

